# Modification of the optical and structural properties of ZnO nanowires by low-energy Ar^+^ ion sputtering

**DOI:** 10.1186/1556-276X-8-162

**Published:** 2013-04-09

**Authors:** Rabie Fath Allah, Teresa Ben, David González, Vanesa Hortelano, Oscar Martínez, Jose Luis Plaza

**Affiliations:** 1 Departamento de Ciencia de los Materiales e Ingeniería Metalúrgica y Q.I., Facultad de Ciencias, Apdo. 40, Puerto Real, Cádiz, 11510, Spain; 2 GdS-Optronlab Group, Departamento Física Materia Condensada, Edificio I+D, Universidad de Valladolid, Paseo de Belén 1, Valladolid, 47011, Spain; 3 Laboratorio de Crecimiento de Cristales, Departamento de Física de Materiales, Facultad de Ciencias, Universidad Autónoma de Madrid, Cantoblanco Madrid, 28049, Spain

**Keywords:** Irradiation, ZnO, Nanowires, 81.07.Gf nanowires, 68.37.Lp transmission electron microscopy, 61.80.Lj irradiation effects, 78.66.Jg optical properties, 81.40.Vw structural properties of materials.

## Abstract

The effects of low-energy (≤2 kV) Ar^+^ irradiation on the optical and structural properties of zinc oxide (ZnO) nanowires (NWs) grown by a simple and cost-effective low-temperature technique were investigated. Both photoluminescence spectra from ZnO NW-coated films and cathodoluminescence analysis of individual ZnO NWs demonstrated obvious evidences of ultraviolet/visible luminescent enhancement with respect to irradiation fluence. Annihilation of the thinner ZnO NWs after the ion bombardment was appreciated by means of high-resolution scanning electron microscopy and transmission electron microscopy (TEM), which results in an increasing NW mean diameter for increasing irradiation fluences. Corresponding structural analysis by TEM pointed out not only significant changes in the morphology but also in the microstructure of these NWs, revealing certain radiation-sensitive behavior. The possible mechanisms accounting for the decrease of the deep-level emissions in the NWs with the increasing irradiation fluences are discussed according to their structural modifications.

## Background

The outstanding and novel physical properties determined in zinc oxide (ZnO) nanowire (NW) special shapes and structures are the reason for which nanoscale one-dimensional semiconductor materials have attracted much attention in recent years [1]. ZnO NWs are very promising as a consequence of their direct bandgap of 3.37 eV (at room temperature) and an exciton binding energy, 60 meV, larger than their thermal energy at room temperature (RT) that enables the observation of excitonic emission at RT. Because of this, they can be used for a wide range of applications such as ultraviolet (UV) light-emitting devices [2], nanogenerators [3], rectifying diodes [4], sensors [5], and electron emitters [6].

Many techniques offer the possibility to obtain ZnO NWs, such as metal-organic chemical vapor deposition, vapor phase epitaxy, direct carbo-thermal growth, and pulsed laser deposition [7,8]. However, all these techniques require low pressures and high operating temperatures (800°C to 1,400°C). Recently, the hydrothermal synthesis route has been successfully applied to the growth of ZnO nanostructures at lower temperature [9-12]. However, despite its easy implementation, the growth rate [13] and the optical quality of the resultant as-grown ZnO nanostructures are generally poorer than those grown by the other techniques, and the technique offers low reproducibility (size and shape control) [14,15]. Our group and other researchers have already reported on the successful growth of high-quality ZnO NWs using a simple technique consisting in the oxidation of Zn metal films in ambient conditions [16-22]. The simplicity of the process, the low temperature required (close to 500°C), as well as the good quality of the obtained NWs make this method attractive for future nanodevice applications.

It is noteworthy that many reports on the optical properties of ZnO nanorods and NWs point out to the apparition of a deep-level emission (DLE) band in the visible, together with the near-band edge emission (NBE) in the UV. In this sense, to change their optical properties, several studies on emission tailoring of ZnO NWs exposed to an irradiation source have already been developed [23-25] but with contradictory outcomes. In particular, with regard to the optical response, Krishna and co-workers reported the occurrence of several bands in the visible region which were identified in the PL spectra of 15-keV energy Ar^+^-irradiated thin films. They indicated a strong detraction of the visible signal with respect to the UV emission [26], and similar optical results were confirmed by Liao and co-authors in the case of 5 to 10 kV Ti-implanted ZnO NWs [27]. Besides the modification of the UV/visible intensity ratio, UV signal blueshift was found by Panigrahy for 2- to 5-keV Ar^+^-irradiated ZnO nanorods [28]. The UV blueshift was also detected in the cathodoluminescence (CL) spectra of ZnO NWs irradiated with 30-keV Ti^+^ ions. Nevertheless, in this case, the visible emission did not suffered changes with the implantation doses [29], contrary to the behavior observed by Wang et al. [30] who reported a complete disappearance of the visible emission from ZnO NWs irradiated with 2-keV H^+^ ions. Hence, the modification of the luminescence properties of ZnO after irradiation experiments is still not clearly understood and, even less, after low energy irradiation experiments. In any case, it would be desired to tailor the ZnO NW emission by minimizing the visible emission and therefore improving the UV luminescence. This would be particularly important in the case of cost-effective growth procedures, for which the obtained ZnO NWs could present some important emissions in this spectral range.

In this work, we present the results of exposing ZnO NWs to a low-energy (≤2 kV) Ar^+^ ion irradiation. These experiments require a relatively simple experimental setup where only a small high-vacuum chamber and an ion gun are needed. Our experimental results show that the irradiation gives rise to an increase of the UV emission with respect to the visible one. We base the explanation of these effects on the structural analysis performed on individual NWs. From these results, we conclude that low-energy Ar^+^ ion irradiation is a promising method to tailor the luminescent properties of ZnO NWs.

## Methods

For the growth of the ZnO NWs, LiNbO_3_ (LN) substrates were chosen, motivated first by the absence of interaction between the substrate (LN) and the ZnO films, demonstrated in our previous unpublished experiments, and second, the suitability of the LN/ZnO system for the development of various applications such as surface acoustic wave gas sensor devices [31,32]. The *c*-axis-oriented LN substrates used in this work were grown in our laboratory by the standard Czochralski technique. LN substrates of about 1 mm thick were cut perpendicular to the *c*-axis. A Zn metal film was evaporated at 800°C on top of the LN substrates. The evaporation took place for 5 min inside a quartz ampoule located in a horizontal furnace. Only the Zn (6N), 0.5755 g, pellets were heated, keeping the LN substrate close to RT during this evaporation step. A further oxidation step was performed in air at 500°C. This process was stopped after about 23 h, when the Zn film thickness reached values near to 30 μm, as deduced by means of profilometry measurements. This technique has already been successfully used to grow high-quality ZnO NWs on other substrates such as CdTe [18]. The obtained NWs grow on top of the ZnO films formed by the oxidation of the Zn film evaporated layer. More details of the preparation technique can be found elsewhere [18]. After confirming the formation of a quite homogenous NW cover layer on the sample, several areas were independently irradiated with different Ar^+^ ion beam fluences. The Ar^+^ irradiation took place inside a home-made high-vacuum (10^−6^ mbar) chamber system equipped with a Specs IQE-11 broad beam ion gun (Berlin, Germany). Irradiation energies of 500 and 2,000 V were used, which result in fluences of 1.5 × 10^16^ cm^−2^ and 10^17^ cm^−2^, respectively (the irradiation time was always 1 h).

High-resolution scanning electron microscopy (HR-SEM) analyses were carried out by using a Philips SEM-FEG-XL30 microscope (Amsterdam, the Netherlands). Energy-dispersive X-ray in SEM mode (EDX-SEM) analysis was performed in a SEM microscope (Hitachi S-3000 N, Chiyoda, Tokyo, Japan), with an attached EDX analyzer (Oxford Instruments, model INCAxsight, Abingdon, Oxfordshire, UK). CL measurements were carried out at liquid nitrogen temperature (80 K) using a XiCLone (Gatan, UK) module attached to a LEO 1530-Carl Zeiss-FESEM microscope (Oberkochen, Germany). The luminescence signal was detected with a Peltier-cooled CCD (Photometrics Ltd., Tucson, AZ, USA). Micro-photoluminescence (μPL) measurements at RT were obtained with a HRLabRam spectrometer (HORIBA Jobin Yvon Inc., Edison, NJ, USA) attached to a metallographic microscope. The excitation was done with a He-Cd laser line at 325 nm, through a ×40 microscope objective, which also collected the scattered light. Conventional transmission electron microscopy (CTEM) and high-resolution transmission electron microscopy (HR-TEM), as well as EDX spectroscopy in scanning transmission electron microscopy (TEM) mode, were realized using a JEOL 1200 EX (JEOL Ltd., Akishima, Tokyo, Japan) and a 2010 F microscope operating at 120 and 200 kV, respectively. The latter is equipped with an Oxford Instruments’ EDX detector. For these measurements, the NWs were scraped from the substrate and dispersed on a lacey carbon-coated copper grid.

## Results and discussion

**Figure 1:**
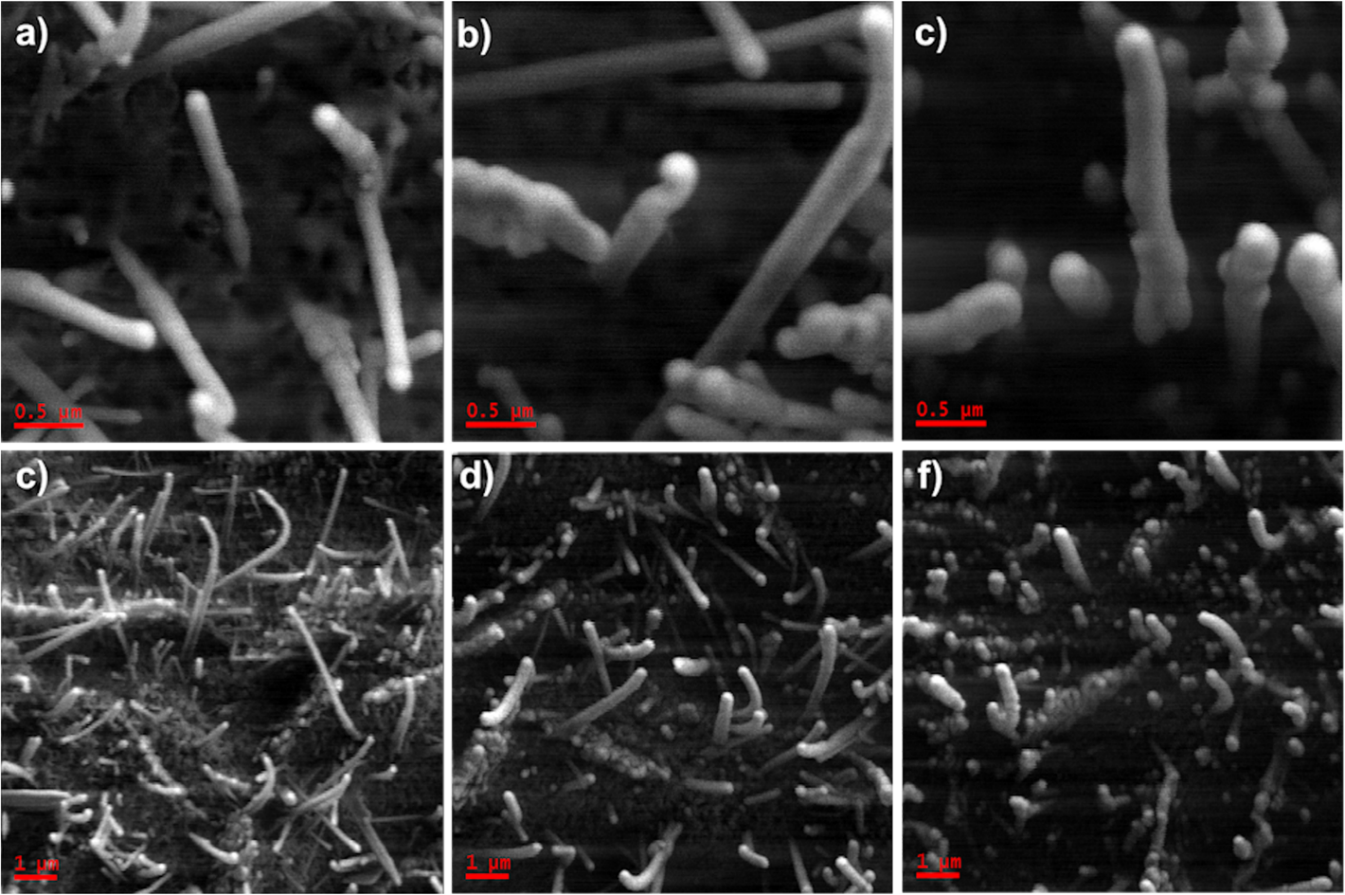
**High-resolution SEM images.** Showing the morphology of unirradiated ZnO NWs (**a**, **b**) and irradiated NWs with fluences of 1.5 × 10^16^ cm^−2^ (**c**, **d**) and 10^17^ cm^−2^ (**e**, **f**). Note the disappearance of the thinner NWs as the irradiation fluence increases.

Just after growing the NWs and before performing any irradiation, EDX-SEM analysis (not shown here, see Additional file 1) confirmed that the ZnO film composition was very close to the stoichiometric one (O 50.50%, Zn 49.5%). In order to determine if the irradiation could affect the ZnO NW morphology, HR-SEM analyses were performed. Figure 1a,b shows the SEM images from as-grown unirradiated NWs, with the presence of a quite homogeneous ZnO NW cover layer on top of the ZnO film. Noticeable morphology changes can be observed on the surfaces of the films after irradiation (Figure 1c,d) where the images evidence a reduction of the thinner ZnO NW population, and only relatively thicker NWs can be observed. This is still more evident for the highest fluence (Figure 1e,f). Thus, it can be concluded that, at least for the fluences used in this work, the thinner NWs (diameter (*d*) < 200 nm) do not survive the irradiation process, especially at higher fluences (10^17^ cm^−2^). In addition, the remaining NWs seem increasingly thicker when the irradiation fluence increases.

Before any structural or optical characterization, the irradiated areas were observed by the naked eye when illuminating under UV light (at 365 and 254 nm). A clear color change was detected with respect to the unirradiated areas; the irradiated ones appear black (not shown here, see Additional file 2). This was the first evidence of an important change in the optical emission properties of the samples, which motivated a detailed optical characterization of the irradiated structures.

**Figure 2:**
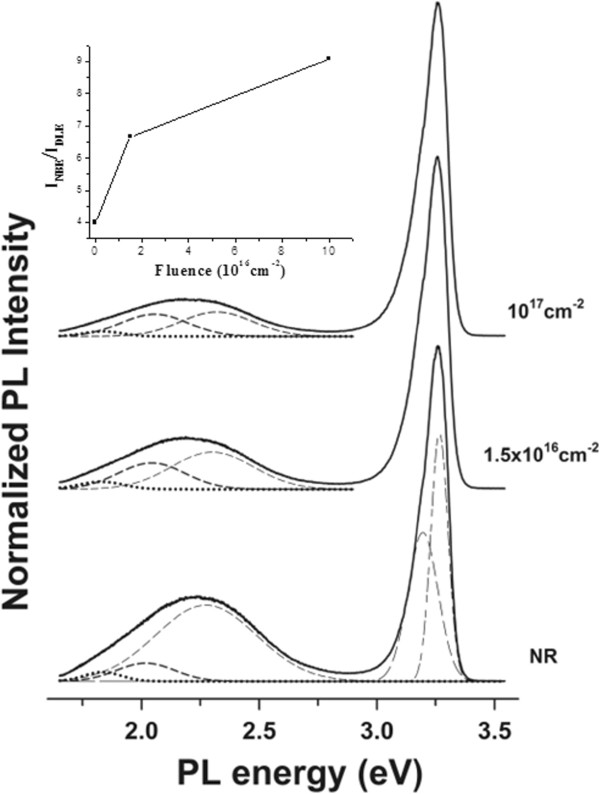
**μPL spectra.** Unirradiated (NR) and irradiated areas with fluences of 1.5 × 10^16^ cm^−2^ and 10^17^ cm^−2^. The spectra, normalized to the band-to-band recombination, show the diminution of the visible band intensity as the irradiation energy increases. Gaussian deconvolution bands are also shown. The inset shows the intensity ratio *I*_NBE_/*I*_DLE_ as a function of the irradiation fluence.

For a more in-depth study, μPL measurements were performed at RT on both the unirradiated and irradiated areas (Figure 2). The two typical emissions of ZnO were always observed, a strong NBE UV emission (approximately 3.26 eV) due to the direct recombination of photogenerated charge carriers or excitons [33] and a broad visible emission band (approximately 2.25 eV) involving deep levels. It is proposed that the visible emission (DLE) in ZnO originates from the contribution of at least three subbands, i.e., the so-called green band (green luminescence (GL), at approximately 2.4 eV (approximately 515 nm)), the yellow band (yellow luminescence (YL), at approximately 2.1 eV (approximately 590 nm)), and the orange luminescence (OL)-red luminescence (RL) (at approximately 1.9 to 2.0 eV (620 to 652 nm) and 1.8 to 1.9 eV (652 to 690 nm), respectively). The relative intensity of these bands depends on the sample preparation method. The GL has been mainly associated with oxygen vacancies, *V*_O_[34-38]. Zn deficiency-related defects (zinc vacancies, *V*_Zn_, oxygen in Zn positions or antisites, O_Zn_, or oxygen interstitials, O_i_) have been proposed as the origin of the yellow and orange-red luminescence emissions [39,40], while impurities (mainly Fe) have been claimed as responsible for the RL [41]. However, there are important discrepancies in the assignation of the origin of the visible contributions, being still a matter of high controversy [42].

The deconvolution of the visible bands gives two main contributions at 2.05 and 2.30 eV - a residual contribution at 1.83 eV is also observed - being 2.30 eV as the predominant one (see Figure 2). The spectral position of these bands would indicate a contribution from both the GL and the YL emissions. As we can see in the figure, the irradiation seems to affect mainly the GL emissions with a strong reduction of this contribution with the increase of the fluence. Consequently, a tiny redshift is observed in the broad band of the visible emission. Normalizing the NBE emission band, it is observed that the ratio between the NBE and visible emissions increases in the irradiated areas, the increase being more pronounced when the irradiation fluence increases. Thus, the low-energy (≤2 kV) Ar^+^ irradiation brings about a rearrangement of the ZnO lattice with a reduction of the DLE and a relative increase of the NBE transition (excitons).

**Figure 3:**
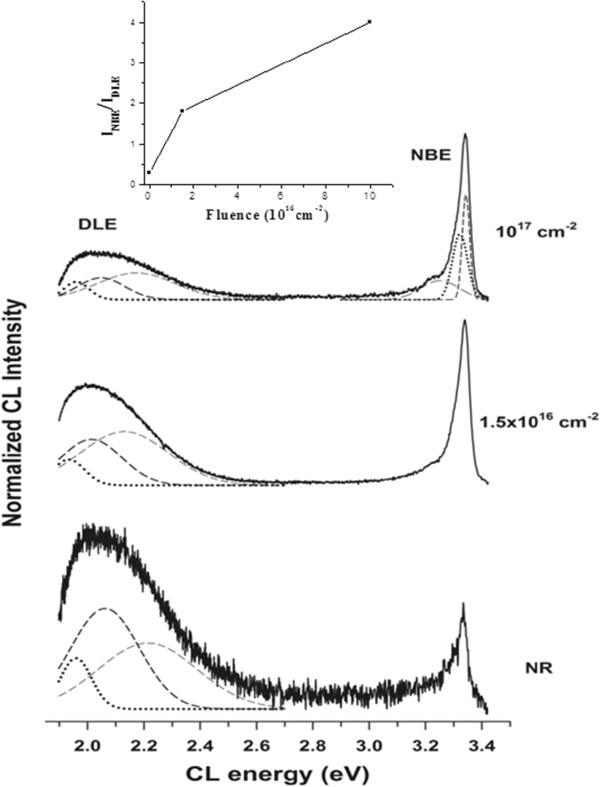
**Normalized CL spectra collected on individual NWs.** Unirradiated (NR) and irradiated areas with fluences of 1.5 × 10^16^ cm^−2^ and 10^17^ cm^−2^. An increase of the NBE emission with respect to the visible band as the irradiation fluence increases is observed (see the inset). Gaussian deconvolution bands are also shown.

To study the specific properties of individual ZnO NWs, CL measurements with high spatial resolution of individual NWs with similar dimensions were also performed on both unirradiated and irradiated areas (Figure 3). It is observed that a rebalance between the NBE and visible emissions on the NWs with the increase of the irradiation fluence occurs. The intensity ratio NBE/DLE is amplified (see the inset) changing from a value of approximately 0.3 in the unirradiated areas to a value of approximately 4 for the sample irradiated with a fluence of 10^17^ cm^−2^. This is clear evidence that the irradiation with Ar^+^ ions (even with low energies, ≤2 kV) influences the emission behavior of the ZnO NWs. Comparing these data with the μPL outcomes, some differences can be detected, in particular concerning the visible emission at higher energies. Two predominant emissions at approximately 2.05 and approximately 2.2 eV are observed after deconvoluting the CL luminescence bands. The position of the deconvoluted CL luminescence bands slightly changes with the irradiation. The two main contributions are situated at 2.06 and 2.21 eV for the NR sample, at 2.01 and 2.13 eV for the sample irradiated with an intermediate fluence, and at 2.05 and 2.17 eV for the sample irradiated with the highest one. As mentioned, there is an important diminution of the whole visible band with respect to the NBE emission with the irradiation process, especially the diminution of the 2.05 eV contribution. A residual additional band at 1.96 eV, deduced from the convolution process, remains nearly without changes.

The differences in the observed luminescence bands between μPL and CL spectra can be a consequence of the different excitation conditions used in both kinds of measurements. Indeed, some authors have reported noticeable differences in the shape of the visible band in ZnO NWs depending on the PL excitation conditions [43]. Since the relative intensity of the defect emission bands can be significantly affected by the excitation power conditions and taking into account the controversial results reported in the literature for the different contributions (GL, YL, and RL) [42], caution needs to be taken to assign an exact origin for the DLEs in our NWs as well as to explain the changes observed between the μPL and CL results. From all these considerations, the main conclusion from our analysis is the diminution of the DLE with respect to the NBE in the NWs with the increase of the irradiation fluence.

**Figure 4:**
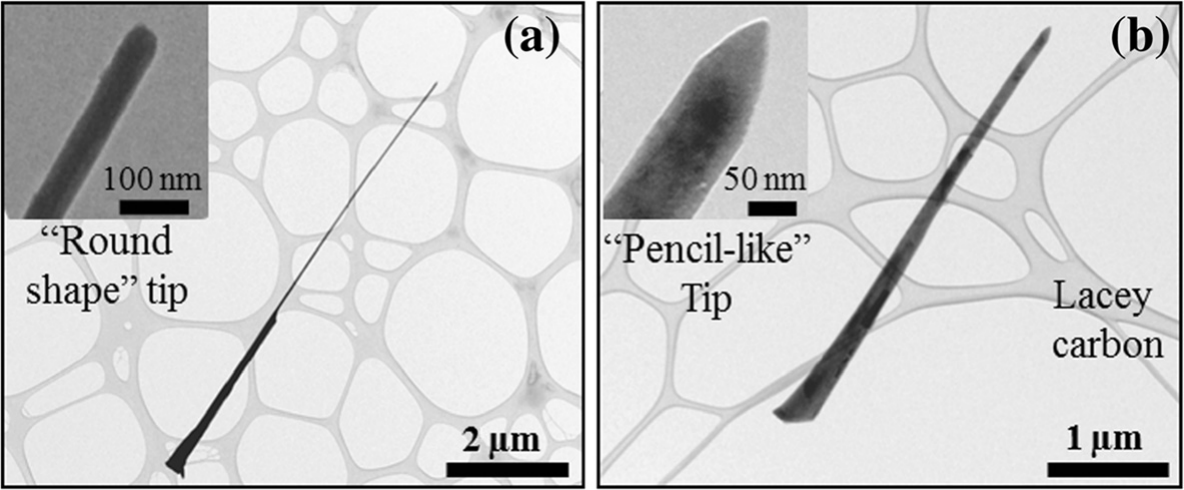
**CTEM images showing two representative ZnO NWs (a, b).** Extracted from unirradiated and irradiated (fluence = 10^17^ cm^−2^) areas, respectively. The insets of both figures show the nanowire tip details; note that the irradiated NW tip is faceted as a consequence of the strike by Ar^+^ energetic particles.

**Figure 5:**
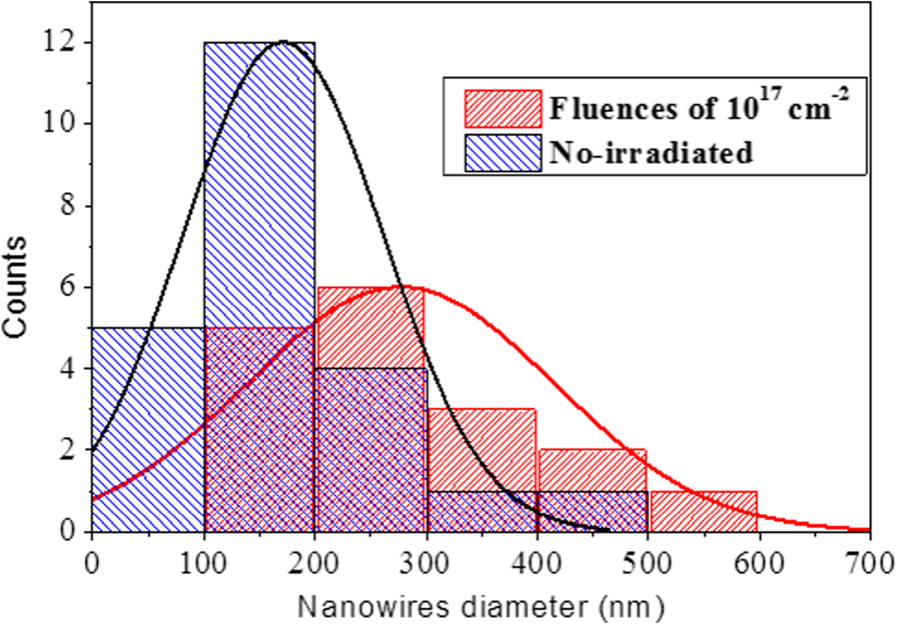
**Diameter distribution in the lower part of nanowires.** Scraped from both the unirradiated and irradiated (fluence = 10^17^ cm^−2^) areas. The NW diameter NW frequency increases for the latter case.

Characterization by suitable techniques to understand the correlation between structural and optical properties is of particular interest. For this purpose, morphological and structural measurements of individual ZnO NWs have been performed by CTEM and HR-TEM techniques and compared with the optical results. Figure 4a,b shows TEM images of two representative ZnO NWs extracted from an unirradiated and 2-kV irradiated area, respectively. Due to their common origin, any morphological changes between them must be related to the irradiation process (assuming a similar morphology of as-grown NWs, according to the observed NWs in the unirradiated areas). From the CTEM images, the NWs from the unirradiated areas seem to be formed by two regions with different diameters: a relatively conical base which sharpens up to a certain height and over it a top section with relatively constant radius. However, most of the 2-kV irradiated wires seem to lose the upper thinner region exhibiting a conical shape with a homogeneous but strong diameter decrease (see Figure 4b). A statistical study of the diameters of the ZnO NWs was performed in order to determine differences in the NW population due to irradiation. The plot in Figure 5 displays the histogram of the NW base diameter for both cases. It highlights the loss of thinner NW families (with diameters lower than 200 nm) as a consequence of Ar^+^ irradiation, and revealed a better resistance of wider ZnO NWs to the irradiation as a consequence of their lower surface/volume ratio. As a consequence, we noticed an increase of the thicker irradiated NW frequency (*d* > 200 nm) compared to the unirradiated ones, which was in agreement with HR-SEM observations. Similar behavior occurs with regard to the NW length. All the morphological changes can be explained considering the effect of the Ar^+^ ion impinging on the NWs and the progressive annihilation of thinner ZnO NWs, an effect that is reinforced as the irradiation fluence is increased. During the irradiation, the upper parts of the NWs suffer more morphological changes than the lower shadowed parts and in some cases even disappear. The additional formation of ‘pencil-like’ (inset of Figure 4b) tip shapes, only observed in irradiated wires, confirms these later ideas.

**Figure 6:**
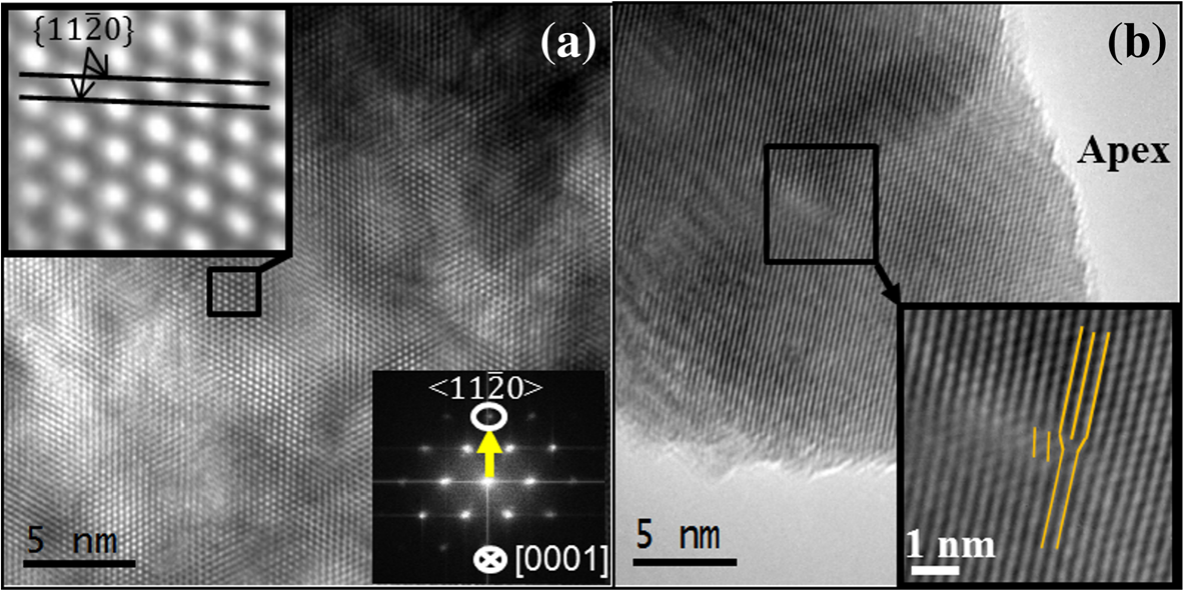
**HR-TEM images of ZnO NW.** (**a**) HR-TEM image recorded on an irradiated ZnO NW (fluence = 10^17^ cm^−2^) confirming the high crystalline quality of the nanowire; the inset shows the corresponding FFT recorded along the [0001] zone axis. (**b**) HR-TEM micrograph of one individual irradiated ZnO NW (fluence = 10^17^ cm^−2^) faceted tip. The inset corresponds to the small squared region of the tip, showing the appearance of one extra plane (edge dislocation).

**Figure 7:**
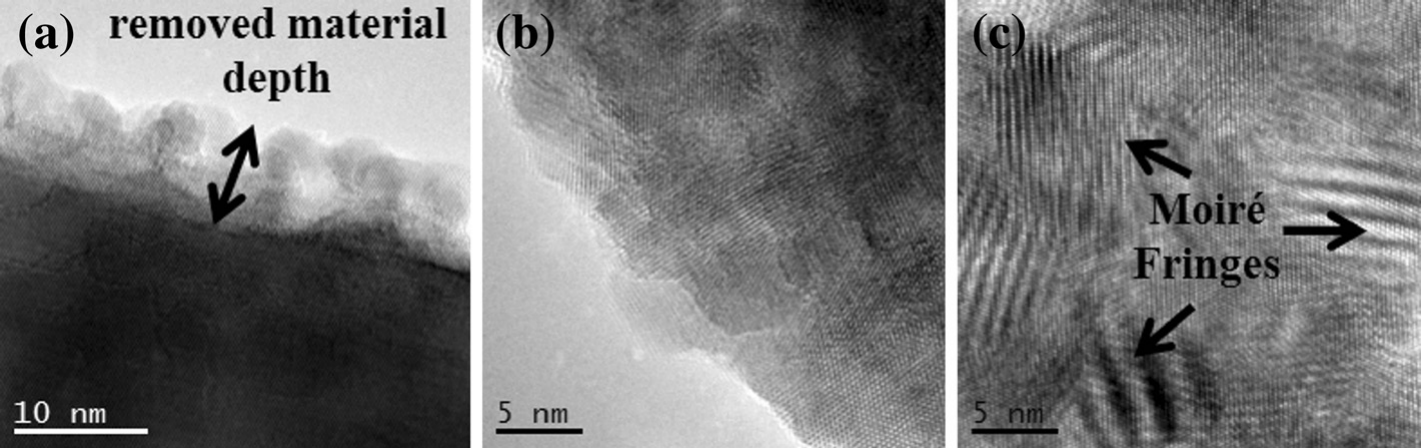
**HR-TEM micrographs of ZnO nanowires irradiated with a fluence of 10**^**17 **^**cm**^**−2**^**.** Showing (**a**) an example of the etched surface (in this case, the removed material layer depth is about 10 nm). In (**b**, **c**), redeposited crystalline particles, with different orientations in the cross-sectional surface and the inner region of the wire, respectively, are observed.

It is well known that the damage level expected for an irradiation process in nanometric materials is much higher than in the bulk due to a larger surface-to-volume ratio, which can induce surface modifications and defect cluster formation. However, despite the irradiation process, TEM micrographs of our NWs indicate that the amorphization degree for most irradiated areas is minimal, and the ZnO NWs generally preserve their good crystalline quality. Figure 6a is an example of HR-TEM image corresponding to one scraped NW from the area irradiated with the highest fluence (10^17^ cm^−2^), which reveals the single-crystalline nature of the NW grown along the [11-20] direction that is one of the three types of fast growth directions in the ZnO NW generation [44]. The inset shows its corresponding fast Fourier transform (FFT), which is consistent with the wurtzite structure of ZnO observed along the [0001] zone axis. Although the high crystalline quality is obvious here and well-defined atomic columns are clearly visible, some ZnO NWs however display stacking faults and dislocations, as well as no well-defined boundaries when observing the wire surface. Such structural modifications are results of preferential bombardment in determined areas of the wires, as can be observed in the NW tip presented in Figure 6b. Regarding the irradiation effect, the ion energies used here are much lower compared to those reported in the bibliography [45]. However, structural changes in ZnO NWs are induced, and the sensibility of some of their properties to low-energy ion irradiation is revealed. The defects found here can be considered as a result of the precipitation of point defects generated during the irradiation. Although defect formation and surface roughness are usual in the irradiated NWs, some NWs undergo higher modifications induced by the Ar^+^ irradiation. Thus, HR-TEM studies revealed that some of the irradiated ZnO NWs were surrounded by a degraded sheath with the same crystalline orientation of the NW core (Figure 7a). Spots shown in the FFT images from these superficial structures were correlated with the inter-planar distances of ZnO. In the extreme case, other irradiated ZnO NWs are surrounded by crystalline nanoparticles with the same ZnO structure but with different orientations with respect to the core (Figure 7b,c), causing the formation of moiré fringes generated by the overlapping of the nanoparticle and NW lattices. In addition, the compositional analysis carried out by EDX spectroscopy (not shown here, see Additional file 3) confirmed that the superficial structures were made up of ZnO. The origin of this sheath is unclear, but it could be the after effect of the sputtering process due to the Ar^+^ impingement. Taking into account all the above data, it can be concluded that the ZnO removed from near the surface of the NWs or even from the annihilation of thinner NWs could sublimate and finally be re-deposited on the remaining NWs giving rise to a core/shell structure of a single ZnO crystal NW core surrounded by a ZnO polycrystalline shell. In addition, the possibility of zinc segregation in our irradiated samples cannot be excluded either. The formation of adatoms on the surface after the irradiation is possible [46], and this surface can grow by the agglomeration of the engendered adatoms during the early stages of bombardment.

**Figure 8:**
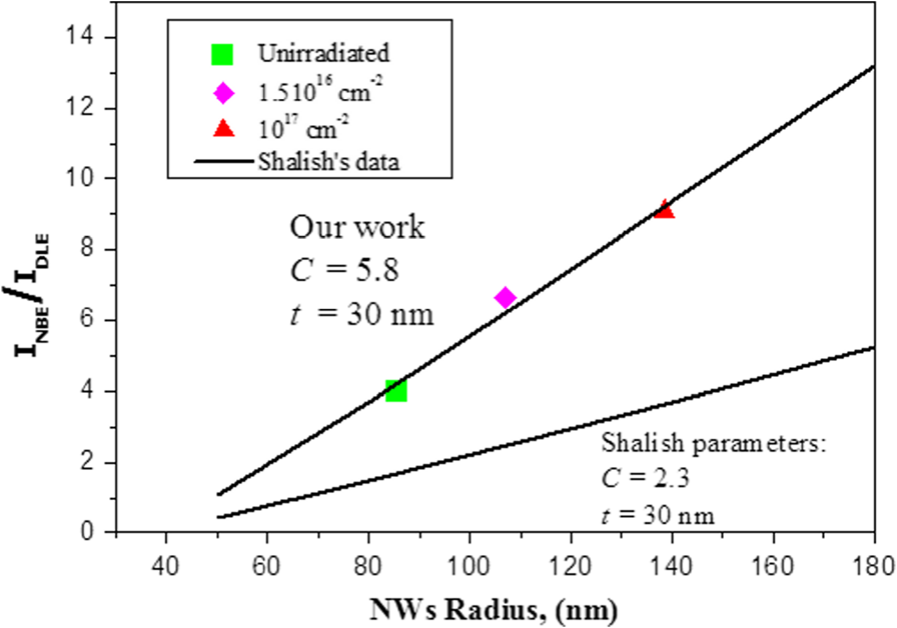
**Experimental luminescence peak intensity *I***_**NBE**_**/*I***_**DLE **_**as a function of the average wire radius.** Values predicted by Shalish’s data are also included.

Taking into account the above structural results, the following possible scenario to account for the effect of the irradiation process on our ZnO NWs can be proposed: Firstly, the ion impinging on the NWs seem to eliminate the thinner NWs, giving rise to an increase of the thicker (*d* > 200 nm) irradiated NW frequency compared to the unirradiated ones (erosion effect due to Ar^+^ ions impinging in the sample). This modification of the NW diameter distribution affects the luminescence properties of the ZnO NWs changing the contribution of the surface luminescence regarding the band edge emission. Shalish et al. [47] observed that the relative intensity of the UV photoluminescence peak was stronger, and the visible luminescence becomes relatively weak as the size of ZnO NWs increases. They explained this size effect in terms of bulk-related to surface-related material-volume ratio, assuming a surface layer thickness, *t*, wherein the surface recombination probability is 1 [47]. The intensity ratio defined by Shalish is as follows:

(1) $$\frac{I_{\text{NBE}}}{I_{\text{DLE}}}=\frac{C{\left(r-t\right)}^{2}}{t\left(2r-t\right)},$$

where *C* is a fitting parameter accounting for the efficiency of the bulk-related emission process relative to the surface and *r* is the wire radius. The UV-visible luminescence intensity ratios (*I*_NBE_*/I*_DLE_) calculated in our samples from the PL curves of Figure 2 are presented in Figure 8 as a function of the average wire radius (deduced from the C-TEM statistical analysis). In our case, the best fit is obtained with *C* = 5.8 and *t* = 30 nm, and Figure 8 also includes data from Shalish et al. using *C* = 2.3 and *t* = 30 nm. The trend in both is very similar with the same surface layer thickness, i.e. an intensification of the UV/visible ratio as the wire diameter increases. The ratio exhibits a clear escalation for thicker NWs (6.6 and 9 for the irradiated NWs with fluences of 1.5 × 10^16^ cm^−2^ and 10^17^ cm^−2^, respectively). The differences of the *C* parameter (between our results and those of Shalish) only mean that the efficiency of the bulk-related emission process regarding the surface is higher in our case. Those discrepancies can be explained by the fact that the compared NWs have been grown by different methods and undergone different treatments, and therefore, it is expected that they initially present different luminescence characteristics since surface state densities are notorious for their great variability.

Nevertheless, if the visible emission is supposed to be mainly originated from defects related to the surface, other factors apart from the annihilation of the thinnest NWs might also be considered. Both μPL and CL data reveal an enhancement of the UV/visible ratio with the increase of the irradiation fluence. Certainly, a reduction of the point defect density in the surface would also result in the UV emission enhancement as a consequence of a net reduction of the visible emission. Perhaps, surface diffusion and annealing effects due to a temperature increase induced during the irradiation process could favor it in some way [48]. However, it is important to mention that the thermal changes near the sample surface were measured during the irradiation processes by a thermocouple installed in the sample holder inside the irradiation chamber. The temperature of the sample only increase up to 60°C during the irradiation, so it is not expected that thermal changes deeply affect to the point defect removal.

It is more likely that the irradiation process can activate a point defect movement, giving rise to a close pair recombination by point defect migration. These diffusion processes have also been known to have important effects on the surface structure, even inducing nanopatterning after low-energy ion irradiation [49,50]. Hence, the effect of the Ar^+^ ions can cause the displacement of Zn atoms from their sites either when they are located as native interstitials or in their equilibrium positions inside the ZnO lattice. This is due to their lower displacement energy compared to that of the oxygen atoms (energy displacement of Zn and O are 18.5 and 41.4 eV, respectively) [51]. Additionally, part of the Zn removed would subsequently segregate towards the surface, favored by their high mobility even at RT [52,53], contributing to the shell structure observed in the HR-TEM images. Indeed, other authors have also reported such Zn segregation to the surface due to the irradiation process, accompanied by a color change [54]; the latter is in agreement with our observations with the naked eye under UV illumination. In our case, we have not detected the presence of metallic Zn even if the color change was evident; these results may not be too surprising taking into account the strong Zn tendency to form oxides when in contact with oxygen, avoiding its TEM observation. Besides, the proposed Zn migration due to the irradiation process can result in a restructuration/reduction of many existing defects, which can effectively passivate deep-level intrinsic defects in the ZnO NWs and consequently decreases the DLE intensity with respect to the NBE emission of the individual NWs. This could explain the increase of the intensity UV/visible ratio showed in the CL spectra where the NWs analyzed (irradiated or not) presented different CL spectra being dimensionally comparable.

Both mechanisms, the annihilation of the thinner NWs and the reduction of defect concentration with the increase of the irradiation fluence, would support the found increase of the intensity ratio between the NBE and the visible emission. Both can work in cooperation and also would explain the good fitting of Shalish’s size-dependent rule and the increase of the *C* parameter. However, further works are needed to clarify the effects of low-energy (≤2 kV) Ar^+^ irradiation on the optical and structural properties of ZnO nanowires.

## Conclusions

Micro-photoluminescence and cathodoluminescence measurements have shown that the irradiation of ZnO NWs with low-energy (≤2 kV) Ar^+^ ions enhances the UV/visible intensity ratio. TEM analysis demonstrated significant changes in the morphology as well as in the microstructure of these NWs, revealing a certain radiation-susceptible nature. HR-TEM studies revealed the loss of thinner NW families and the existence of NWs with surface modifications due to the irradiation with low-energy Ar^+^ ions. We postulate that Ar^+^ ion irradiation would annihilate the thinner ZnO NWs as well as activate Zn diffusion, leading to a restructuration/reduction of many native defects. We attribute the attenuation of the visible emission both to Zn diffusion effect and to the reduction of surface-related volume responsible for the deep-level luminescence. This work demonstrates that an inexpensive technique can improve the luminescent behavior of ZnO NWs grown by a cost-effective technique based on Zn oxidation under low temperature in ambient conditions.

## Abbreviations

CL: Cathodoluminescence; CTEM: Conventional transmission electron microscopy; d: diameter; DLE: Deep-level emission; EDX: Energy-dispersive X-ray; FFT: Fast Fourier transform; GL: Green luminescence; HR-SEM: High-resolution scanning electron microscopy; HR-TEM: High-resolution transmission electron microscopy; LN: LiNbO_3_; NBE: Near-band edge emission; NWs: Nanowires; OL: Orange luminescence; RT: Room temperature; TEM: Transmission electron microscopy; UV: Ultraviolet; YL: Yellow luminescence; μPL: Micro-photoluminescence.

## Competing interests

The authors declare that they have no competing interests.

## Authors’ contributions

JLP designed and grew the samples. OM and VH carried out the PL and CL studies. RFA prepared the TEM samples, acquired the TEM data, and carried out the analysis of results. DG and TB designed the TEM studies and supervised the TEM analysis. All authors actively discussed the results and participated in drafting the manuscript. All authors read and approved the final manuscript.

## Supplementary Material

Additional file 1 **EDX-SEM analysis of ZnO nanowires before the irradiation process.** This file displays a SEM image at low magnification showing the initial sample just after growing the nanowires. On the right of the SEM image, an EDX spectrum is presented with a table containing the quantitative analysis and confirming that the composition was very close to the stoichiometric one. Click here for file

Additional file 2 **Color change detected in ZnO irradiated areas.** This file shows samples irradiated with different energies. As can be seen, a clear color change is observed in the irradiated area by the naked eye when illuminating under UV light. The irradiated areas appear black. Click here for file

Additional file 3 **Compositional analysis carried out by EDX spectroscopy of the superficial particles.** This file presents an EDX spectrum carried out in the superficial particles. The quantitative analysis shown in the table confirms that the superficial particles are made up of ZnO. Click here for file
