# Supplementary figures and images for: Modification of the optical and structural properties of ZnO nanowires by low-energy Ar+ ion sputtering
Source: Nanoscale Res Lett. 2013 Apr 9;8(1):162. doi: 10.1186/1556-276X-8-162 (PMC3648432; doi:10.1186/1556-276X-8-162)

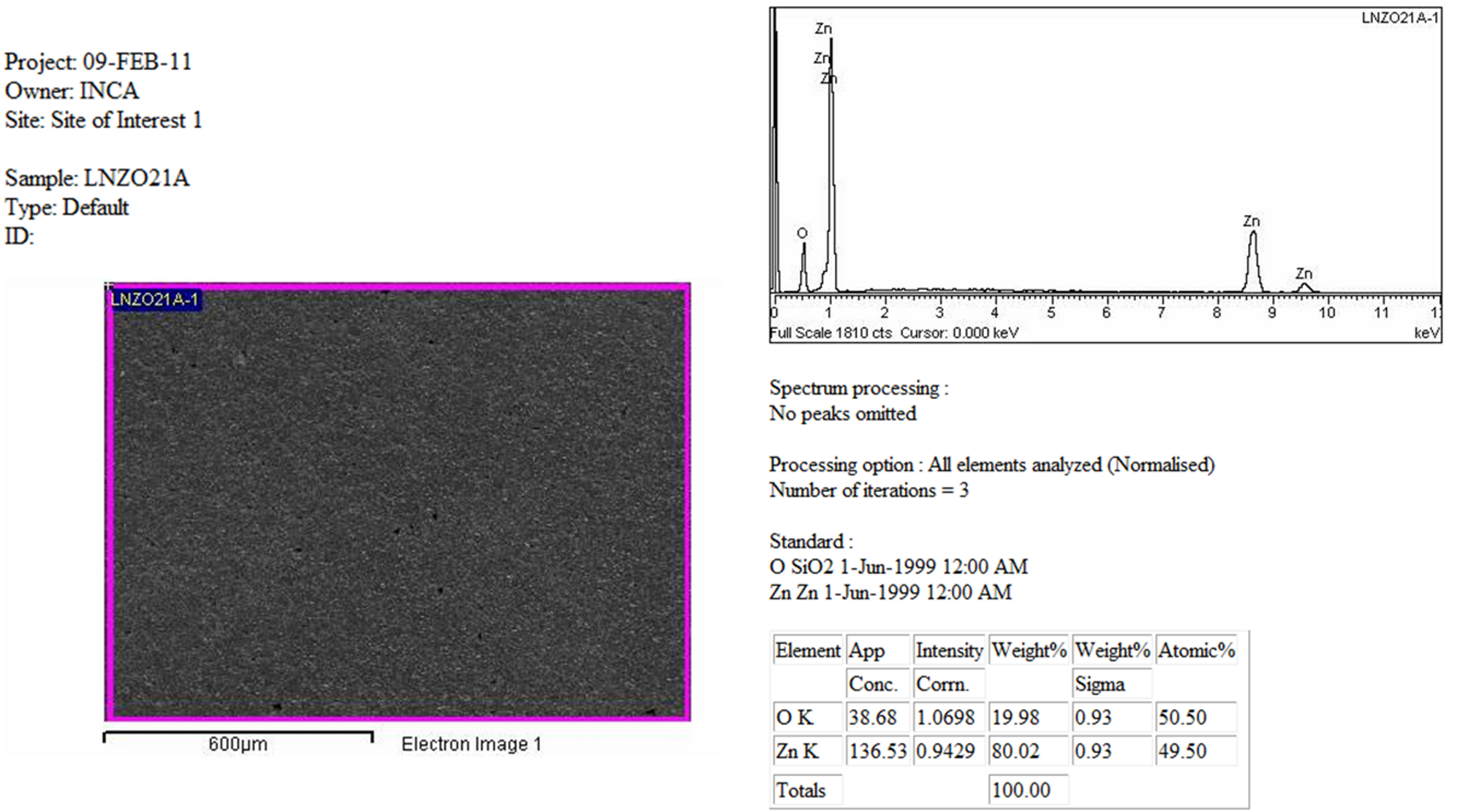

Supplement: Additional file 1 — EDX-SEM analysis of ZnO nanowires before the irradiation process. This file displays a SEM image at low magnification showing the initial sample just after growing the nanowires. On the right of the SEM image, an EDX spectrum is presented with a table containing the quantitative analysis and confirming that the composition was very close to the stoichiometric one. [file 1556-276X-8-162-S1.tiff]

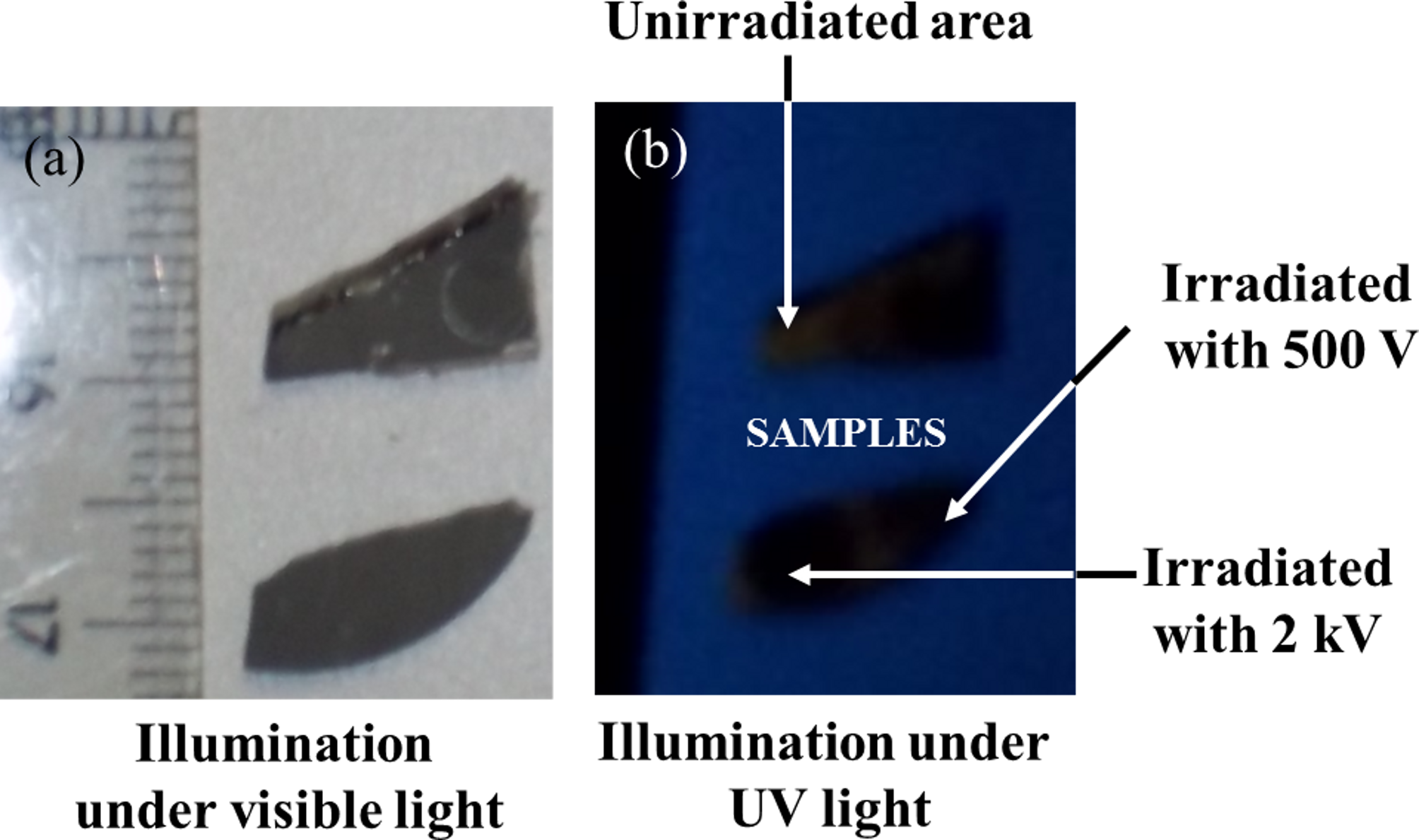

Supplement: Additional file 2 — Color change detected in ZnO irradiated areas. This file shows samples irradiated with different energies. As can be seen, a clear color change is observed in the irradiated area by the naked eye when illuminating under UV light. The irradiated areas appear black. [file 1556-276X-8-162-S2.tiff]

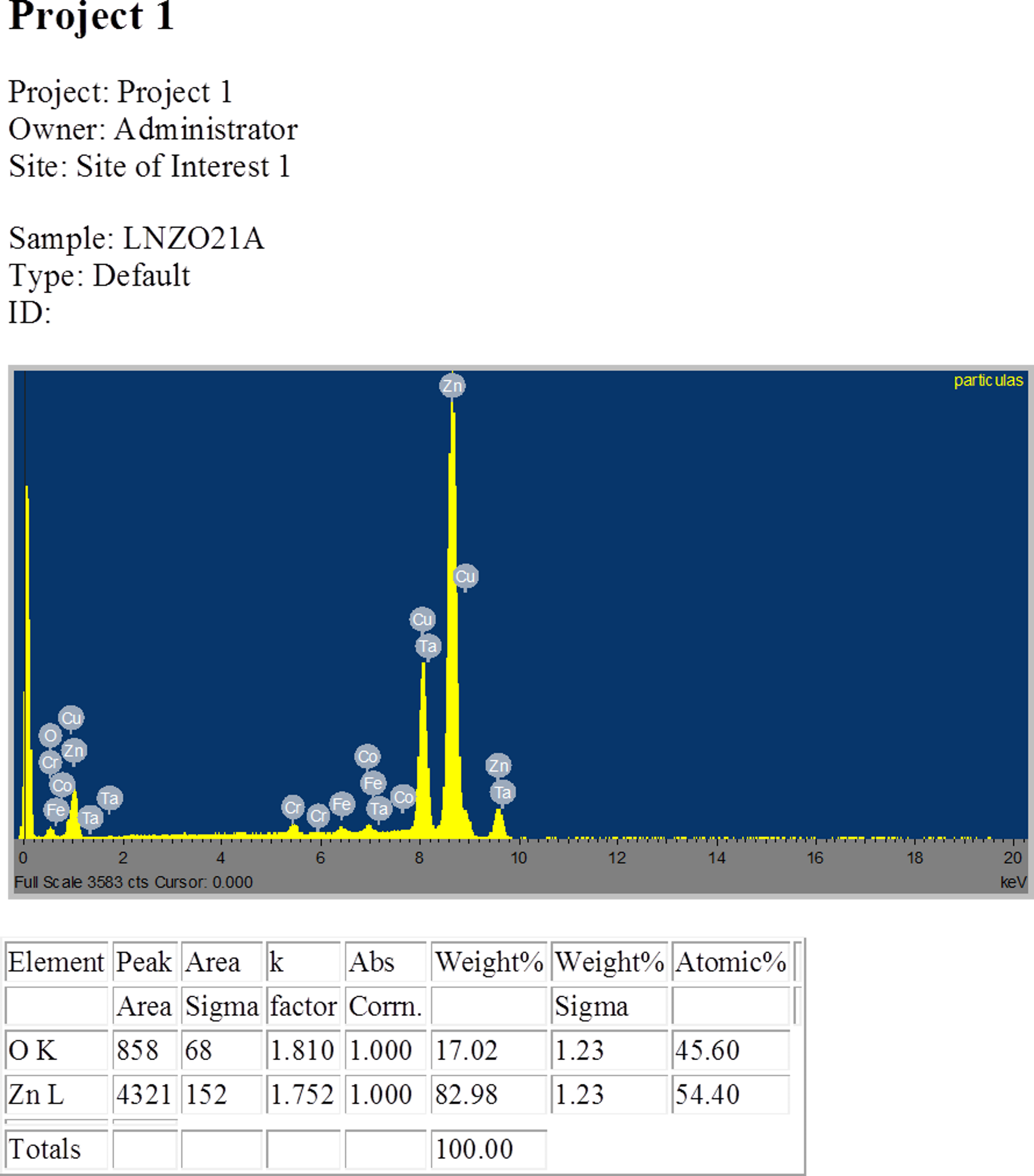

Supplement: Additional file 3 — Compositional analysis carried out by EDX spectroscopy of the superficial particles. This file presents an EDX spectrum carried out in the superficial particles. The quantitative analysis shown in the table confirms that the superficial particles are made up of ZnO. [file 1556-276X-8-162-S3.tiff]
